# PPR Protein BFA2 Is Essential for the Accumulation of the *atpH/F* Transcript in Chloroplasts

**DOI:** 10.3389/fpls.2019.00446

**Published:** 2019-04-12

**Authors:** Lin Zhang, Wen Zhou, Liping Che, Jean-David Rochaix, Congming Lu, Wenjing Li, Lianwei Peng

**Affiliations:** ^1^Shanghai Key Laboratory of Plant Molecular Sciences, College of Life Sciences, Shanghai Normal University, Shanghai, China; ^2^State Key Laboratory of Crop Biology, College of Life Sciences, Shandong Agricultural University, Tai’an, China; ^3^Departments of Molecular Biology and Plant Biology, University of Geneva, Geneva, Switzerland; ^4^College of Life Sciences, Langfang Normal University, Langfang, China

**Keywords:** chloroplast ATP synthase, PPR protein, gene expression, photosynthesis, stability

## Abstract

As a fascinating and complicated nanomotor, chloroplast ATP synthase comprises nine subunits encoded by both the nuclear and plastid genomes. Because of its uneven subunit stoichiometry, biogenesis of ATP synthase and expression of plastid-encoded ATP synthase genes requires assistance by nucleus-encoded factors involved in transcriptional, post-transcriptional, and translational steps. In this study, we report a P-class pentatricopeptide repeat (PPR) protein BFA2 (Biogenesis Factor required for ATP synthase 2) that is essential for accumulation of the dicistronic *atpH/F* transcript in Arabidopsis chloroplasts. A loss-of-function mutation in *BFA2* results in a specific reduction of more than 3/4 of chloroplast ATP synthase, which is likely due to the absence of dicistronic *atpH/F* transcript. BFA2 protein contains 22 putative PPR motifs and exclusively localizes in the chloroplast. Bioinformatics and Electrophoretic Mobility Shift Assays (EMSA) analysis showed that BFA2 binds to the consensus sequence of the *atpF-atpA* intergenic region in a sequence-specific manner. However, translation initiation of the *atpA* was not affected in the *bfa2* mutant. Thus, we propose that the chloroplast PPR protein BFA2 mainly acts as barrier to prevent the *atpH/F* transcript degradation by exoribonucleases by binding to the consensus sequence of the *atpF-atpA* intergenic region.

## Introduction

Chloroplasts in photosynthetic eukaryotes are thought to have originated from cyanobacteria through endosymbiosis. During evolution, most of the genes from the cyanobacterial ancestor were transferred to the nucleus of the host cell and chloroplasts have only retained about 100 genes (Martin et al., 2002). These plastid genes encode proteins required for transcription and translation as well as the essential components of photosynthetic complexes. To ensure efficient gene expression, chloroplasts require a vast number of nuclear-encoded protein factors facilitating transcription, RNA stabilization, splicing, editing, and translation (Stern et al., 2010; Barkan, 2011). Among these factors, pentatricopeptide repeat (PPR) proteins are highly prominent and involved in various steps of RNA metabolism and protein translation (Schmitz-Linneweber and Small, 2008). There are hundreds of PPR proteins in land plants most of which function in chloroplast and mitochondrial gene expression (Barkan and Small, 2014). PPR proteins comprise a large class of proteins with tandem arrays of a 35-amino-acid degenerate motif (Small and Peeters, 2000). According to the PPR motif type, PPR proteins can be divided into two major subfamilies, P and PLS. While P-type PPR proteins contain only P (35 amino acids) motifs with one or more tandem arrays, PLS-class PPR proteins have tandem triplet arrays of P, L (35–36 amino acids), and S (31 amino acids) motifs. Extensive studies showed that the P-class PPR proteins are involved in RNA stabilization, cleavage, and splicing as well as in the activation of translation (Barkan and Small, 2014). A few P-class PPR proteins also contain a small-MutS-related (SMR) motif at their C-terminus, which was recently shown to have RNA endonuclease activity *in vitro* (Zhou et al., 2017). The PLS-class PPR proteins usually contain C-terminal E and DYW motifs which are required for RNA editing (Shikanai, 2015).

Chloroplast ATP synthase is a multi-subunit complex located in the thylakoid membranes. It produces ATP from ADP by utilizing the proton motive force generated by photosynthetic electron transport. Chloroplast ATP synthase is composed of the two CF_o_ and CF_1_ modules, and they contain five and four subunits with the stoichiometry α_3_β_3_γ_1_ε_1_δ_1_ and I_1_II_1_III_14_IV_1_, respectively (Hahn et al., 2018), encoded by both the nuclear and chloroplast genomes. Chloroplast-encoded ATP synthase subunits arise from two polycistronic chloroplast transcription units, the large (*atpI/H/F/A*) and the small (*atpB/E*) *atp* operons. Both operons are transcribed by the plastid-encoded RNA polymerase (PEP) and several sigma factors are required (Malik Ghulam et al., 2012).

During the past decade, several nucleus-encoded factors have been shown to be involved in the expression of *atp* genes. For the large *atp* operon, P-class PPR protein PPR10 binds to the intergenic regions of *atpI-atpH* and *psaJ-rpl33* (Pfalz et al., 2009). The binding of PPR10 to the 5′ end of *atpH* not only stabilizes *atpH* transcripts by blocking 5′→3′ exoribonucleases but also alters the structure of the 5′ end of *atpH* to promote activation of translation initiation (Prikryl et al., 2011). The a*tpF* gene contains a single intron which belongs to the group-II intron family. Splicing of the *atpF* intron requires several protein factors such as CRS1, RNC1, WHY1, WTF1, MatK, and AEF1/MPR25 (Till et al., 2001; Watkins et al., 2007; Prikryl et al., 2008; Kroeger et al., 2009; Zoschke et al., 2010; Yap et al., 2015). Besides splicing, PPR protein AEF1/MPR25 is also required for editing *atpF* RNA in Arabidopsis (Yap et al., 2015). In the chloroplast of *Chlamydomonas reinhardtii*, the TDA1 protein is involved in the trapping and translation activation of *atpA* transcripts (Eberhard et al., 2011). In the case of the small *atp* operon, the PPR-SMR protein ATP4/SVR7 as well as the ATP1 protein have been proposed to be involved in the translation of the *atpB/E* mRNA in maize and Arabidopsis (McCormac and Barkan, 1999; Zoschke et al., 2012, 2013).

In this study, we report the characterization of a chloroplast PPR protein called BFA2 (Biogenesis Factors required for ATP synthase 2) that binds to the *atpF-atpA* intergenic region in a sequence-specific manner. Our results demonstrated that binding of BFA2 to the 3′-UTR of *atpH/F* is essential for stabilization of *atpH/F* RNA.

## Materials and Methods

### Plant Material and Growth Conditions

Arabidopsis plants were grown on soil in the greenhouse (80 μmol photons m^-2^ s^-1^, 16 h photoperiod, 23^o^C) for 3–4 weeks. The *bfa2-1* mutant was isolated from a collection of pSKI015 insertion Arabidopsis lines using the FluorCam imaging fluorometer (FC 800-C, PSI, Czech Republic) (Zhang et al., 2016). The *bfa2-2* mutant (SAIL_571_H02) was obtained from NASC and its T-DNA insertion site was confirmed by genomic PCR and subsequent sequencing of the PCR products. For complementation analysis, a genomic DNA fragment of the *BFA2* gene (3753 bp) was cloned into the pBI121 binary vector. The resulting construct was transformed into *Agrobacterium tumefaciens* C58C and then introduced into *bfa2-1* and *bfa2-2* plants by floral dip transformation.

### RNA Extraction, RNA Blotting, and cRT-PCR Assay

Total RNA was isolated from rosette leaves using TRIzol Reagent (Invitrogen Life Technologies). For RNA blot analyses, a total of 5 μg (for *atpB* and *Actin 7*) or 2.5 μg (for *atpI, atpH, atpE, atpF, atpF* intron, and *atpA*) RNA was fractioned by electrophoresis on 1.5% formaldehyde-containing agarose gels and blotted onto nylon membranes (Hybond-N^+^, GE Healthcare). The RNA was fixed by UV crosslinking (HL-2000 HybriLinker). Pre-hybridization and hybridization were carried out at 50^o^C with the DIG Easy Hyb (Roche) buffer. The probes were amplified from DNA and labeled with digoxigenin-11-dUTP according to the manufacturer’s instructions. Signals were visualized with chemiluminescence analyzer or X-film.

For circular RT-PCR (cRT-PCR) analysis, total RNA was treated with RNase-free DNase I (Takara) to remove the residual DNA before further analysis. 10 μg of total RNA was self-ligated for 2 h at 25^o^C with 10 U of T4 RNA ligase (New England Biolabs). After ligation, RNA was extracted and resuspended in 10 μl of DEPC-treated water. Reverse transcription was performed using 20 pmol of primer and 5 μg of self-ligated RNA for 1 h at 42^o^C with 200 U of M-MLV reverse transcriptase (Thermo). After transcription, 1/20th of cDNA was used in a single PCR amplification reaction and the DNA products were then cloned in the pMD-18T vector for sequencing. The primers used this experiment are listed in Supplementary Table S2.

### Subcellular Localization of GFP Protein

For subcellular localization of GFP protein, the first 200 amino acids (to ensure the complete targeting information of BFA2, the N-terminal 200 amino acids including the first PPR motif were used) of BFA2 were fused in-frame with GFP in the pBI221 vector. The chloroplast and mitochondrial markers were constructed according to Zhang et al. (2016). The resulting constructs were transformed into Arabidopsis protoplasts by PEG-mediated transformation and the protoplasts were placed in darkness for 16 h at 23^o^C. Transient GFP expression was observed using a confocal laser scanning microscope (LSM 510 Meta; Zeiss).

### Electrophoretic Mobility Shift Assays

To express the recombinant BFA2-MBP protein, the cDNA sequence encoding amino acids 62–904 of BFA2 was subcloned into the plasmid pMAL-c5x (New England Biolabs). Expression was induced in *E. coli* BL21 strain with 0.3 mM isopropyl β-D-1-thiogalacopyranoside for 20 h at 16^o^C. Purification of the recombinant protein was performed according to the New England Biolabs protocol. The RNA probe (5′-UAUAGGCAUUAUUUUUUUUUCU-3′, *atpF* sRNA) was chemically synthesized, and its 5′-end was labeled by biotin (Takara Co., Ltd.). For competition assays, a specific probe (nonlabeled *atpF* sRNA) and a nonspecific probe (5′-UUAUGACGAUACUCGGUAGCAUAGAUAUAA-3′; 5′-end of the *ndhA* mRNA) were chemically synthesized.

Recombinant BFA2-MBP was incubated with biotinylated *atpF* sRNA for 30 min at 20^o^C in the binding buffer (10 mM HEPES, pH 7.5, 20 mM KCl, 2 mM MgCl_2_, 1 mM DTT, 5% glycerol, 1 μg tRNA). Subsequently, the reactions were resolved on 6% native polyacrylamide gels containing 2.5% glycerol. The signal was detected with the chemiluminescent detection kit (Thermo, 89880). For competition assays, specific probe and nonspecific probes were added in the reaction buffer.

### Other Methods

Polyclonal antibody against BFA2 was raised in rabbits using the recombinant BFA2 protein (amino acids 62–300 of BFA2). Chlorophyll fluorescence analysis, thylakoid membrane and stromal protein isolation, BN-PAGE, 2D/SDS-PAGE, and immunoblot analysis were performed as previously described (Li et al., 2019). The *g*_H_^+^ was monitored with the Dual PAM-100 according to previously described methods (Rott et al., 2011; Zhang et al., 2018). Polysome association analyses were performed as previously described (Zhang et al., 2018). The rRNAs were stained by Super GelRed (US Everbright Inc., Suzhou, China) and used as fractionation and loading controls. Chloroplast protein labeling and chase was performed as previously described (Zhang et al., 2016, 2018). Immunoblot signals were detected with a Pro-light HRP Chemiluminescent Kit (TIANGEN) and visualized with a LuminoGraph chemiluminescence analyzer (ATTO). Antibodies against CF_1_α (PHY0311), CF_1_β (PHY0312), CF_1_γ (PHY0313), CF_1_ε (PHY0314), CF_1_δ (PHY0315), CF_o_I (PHY0316), CF_o_II (PHY0170S), PsaA (PHY0342), PsaD (PHY0343), D1 (PHY0057), D2 (PHY0060), Cyt *f* (PHY0321), and NdhN (PHY0335) were obtained from PhytoAB (United States).

### Accession Numbers

Sequence data from this article can be found in GenBank/EMBL/DDBJ databases under accession number AtBFA2 (AT4G30825, *Arabidopsis thaliana*), GmBFA2 (Glyma.04G155800, *Glycine max*), OsBFA2 (Os09g25550, *Oryza sativa*), ZmBFA2 (XP_008662784, *Zea mays*), NsBFA2 (XP_009792607.1, *Nicotiana sylvestris*), SbBFA2 (SORBIDRAFT_07g007540, *Sorghum bicolor*), PpBFA2-A (Pp3c16_4140, *Physcomitrella patens*), PpBFA2-B (Pp3c5_2530, *Physcomitrella patens*). The aligned sequences of *atpF-atpA* can be found in the chloroplast genomes of *Arabidopsis thaliana* (At; NC_000932), *Glycine max* (Gm; NC_021650), *Nicotiana sylvestris* (Ns; NC_007500.1), *Oryza sativa* (Os; NC_001320), *Zea mays* (Zm; NC_001666). *Physcomitrella patens* (Pp, NC_005087), and *Selaginella moellendorffii* (Sm, nc_013086).

## Results

### The *bfa2* Mutants Are Defective in Normal Accumulation of the Chloroplast ATP Synthase

**FIGURE 1:**
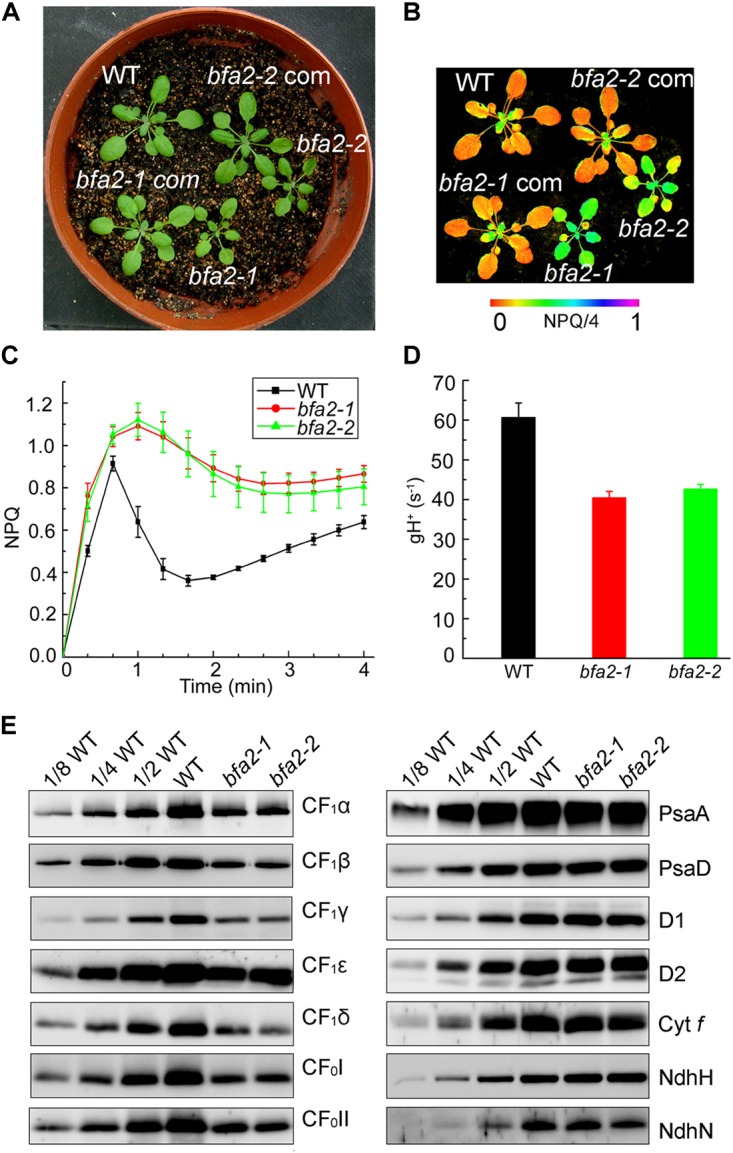
Identification and characterization of the *bfa2* mutants. **(A)** Phenotype of the *bfa2* mutants. *bfa2-1* com and *bfa2-2* com, complemented lines with overexpression of BFA2 in *bfa2-1* and *bfa2-2*, respectively. **(B)** Image of NPQ. The image was captured upon illumination (80 μmol photons m^-2^ s^-1^) for 2 min. Values for NPQ/4 are indicated at the bottom on a false color scale. **(C)** Time course of NPQ induction. NPQ induction kinetics was measured upon illumination with AL (Actinic light) light (80 μmol photons m^-2^ s^-1^) for 4 min. Means ± SD (*n* = 4). **(D)** H^+^ conductivity through ATP synthase (*g*_H_^+^) under an irradiance of 628 μmol photons m^-2^ s^-1^. Means ± SD (*n* = 4). **(E)** Immunoblot analysis of the thylakoid membrane proteins in *bfa2* and WT plants. Proteins were loaded on an equal chlorophyll basis and blots were probed with the indicated antibodies.

While the *bfa2-1* mutant was isolated by screening T-DNA mutant pools (Zhang et al., 2016), *bfa2-2* was obtained from the European Arabidopsis Stock Centre (NASC). Both mutants show high levels of nonphotochemical quenching (NPQ) upon illumination with actinic light (80 μmol photons m^-2^ s^-1^) (Figure 1A,B). During illumination, photosynthetic electron transport induces accumulation of protons in the thylakoid lumen, which persists after illumination for 40 s in the wild-type (WT) plants and triggers the induction of NPQ (Figure 1C). Because of the activation of chloroplast ATP synthase in the light, protons accumulated in the thylakoid lumen move out rapidly through the ATP synthase to produce ATP, resulting in the relaxation of NPQ within 2 min of illumination (Figure 1C). In contrast, the relaxation of NPQ is less efficient in the *bfa2* mutants and NPQ is maintained at high levels compared with WT (Figure 1C). Conductivity of the thylakoids to protons, *g*_H_^+^ (thylakoid conductivity), is usually used to monitor the activity of chloroplast ATP synthase *in vivo* (Cruz et al., 2001). The level of *g*_H_^+^ in *bfa2* is indeed reduced to ∼2/3 of the WT level with an irradiance of 628 μmol photons m^-2^ s^-1^ as actinic light (Figure 1D), implying that the high-NPQ phenotype can be ascribed to the low activity of the chloroplast ATP synthase in *bfa2*.

The seedling size of *bfa2* is smaller than that of WT after germination for 25 days on soil (Figure 1A). To further characterize the phenotype of *bfa2*, several photosynthetic parameters were measured. Fv/Fm, the ratio between variable and maximum fluorescence, that represents the maximum quantum yield of photosystem II (PSII) was found to be comparable between WT and *bfa2* plants (0.79 ± 0.01 for both genotypes), indicating that the function of PSII is not affected. We also investigated the dependence of ETR (electron transport rate through PSII) and NPQ on irradiance. While the ETR is significantly reduced in *bfa2* at an irradiance above 200 μmol photons m^-2^ s^-1^, the level of NPQ is higher in *bfa2* than in WT at all light intensities investigated (Supplementary Figures S1A,B), implying that protons over-accumulate in the thylakoid lumen of *bfa2* and that photosynthetic linear electron transport is inhibited. Analysis of the dependence of 1-qL and the oxidation of the donor side of PSI on irradiance showed that photosynthetic electron transport is significantly restricted between PSII and PSI in *bfa2* compared to WT plants (Supplementary Figures S1C,D). All of these photosynthetic properties in *bfa2* are similar to those of mutants that accumulate low amounts of chloroplast ATP synthase (Zoschke et al., 2012, 2013; Rühle et al., 2014; Fristedt et al., 2015; Grahl et al., 2016; Zhang et al., 2016, 2018).

Immunoblot analysis showed that the levels of the chloroplast ATP synthase subunits in *bfa2* are reduced to ∼25–50% of those of wild-type plants (Figure 1E). In contrast, accumulation of PSI (PsaA and PsaD), PSII (D1 and D2), Cytochrome *b_6_f* (Cyt *f*), and NADH dehydrogenase-like (NDH) complex (NdhH and NdhN) in *bfa2* was as in WT (Figure 1E). Consistent with these results, blue native-PAGE (BN-PAGE) and subsequent two dimensional (2D) SDS-PAGE analysis showed that formation of the NDH-PSI supercomplex, PSII supercomplexes, PSII dimer, PSI monomer and other chlorophyll-containing complexes was not affected in *bfa2* (Supplementary Figure S2A). Although the levels of CF_1_α/β/γ were reduced to about one quarter in *bfa2*, the remaining subunits were assembled into the intact ATP synthase and CF_1_ subcomplex (Supplementary Figure S2B), which accounts for the ∼2/3 activity of ATP synthase in *bfa2* and for its photoautotrophic growth (Figure 1A,D). Taken together, we conclude that accumulation of chloroplast ATP synthase is specifically impaired whereas other thylakoid protein complexes are not affected in *bfa2*. Similar to the *bfa1* and *bfa3* mutants we characterized previously (Zhang et al., 2016, 2018), *bfa2* is also a mutant that accumulates lower amounts of chloroplast ATP synthase.

### BFA2 Is a PPR Protein Present in the Chloroplast Stroma

**FIGURE 2:**
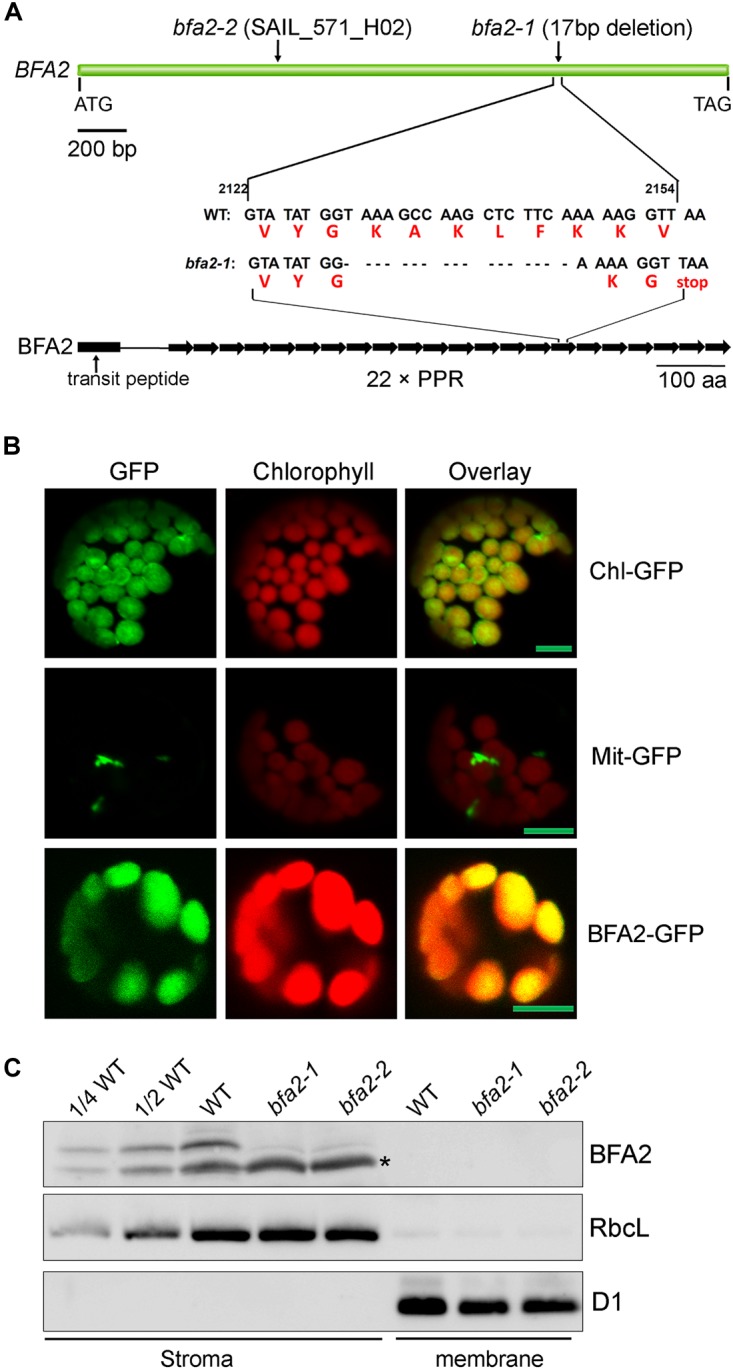
Characterization of the BFA2 protein. **(A)** Schematic representation of *BFA2* gene (Top panel) and BFA2 protein (bottom panel). Positions for nucleotide deletion in *baf2-1* and T-DNA insertion in *bfa2-2* are indicated. Each right arrow represents one PPR domain. The 17-nucleotide deletion results in a premature stop codon at the position of the 16th PPR motif in BFA2. **(B)** Subcellular localization of BFA2 by GFP assay. The first 200 amino acids of BFA2 were fused with GFP (BFA2-GFP) and expressed in Arabidopsis protoplasts. The signal of GFP was visualized using a confocal laser scanning microscope. Chl-GFP and Mit-GFP represent chloroplast and mitochondrial controls, respectively. Bars = 5 μm. **(C)** Immunolocalization of BFA2. Intact chloroplasts isolated from WT and *bfa2* mutants were fractionated into stromal and membrane fractions. Proteins were separated by SDS-PAGE and immunodetected with antibodies against BFA2, RbcL, and D1. The series of WT dilutions is indicated. A major nonspecific band detected in the stromal fractions with BFA2 antibody is indicated by an asterisk. A weak band above the major nonspecific band detected in *bfa2* stroma also appears to be nonspecific.

Map-based cloning detected a 17-nucleotide deletion (2130–2146 bp) in the coding region of *AT4G30825* in *bfa2-1*, resulting in a premature stop codon (Figure 2A). A T-DNA was inserted in the same gene in the *bfa2-2* mutant. Furthermore, overexpression of *AT4G30825* under the control of the *35S* promoter of cauliflower mosaic virus in the *bfa2-1* and *bfa2-2* mutants fully rescued their phenotype (Figure 1A,B). From these results, we conclude that the *AT4G30825* gene corresponds to *BFA2* that is required for full chloroplast ATP synthase activity *in vivo*.

The *BFA2* gene encodes a PPR protein of 904 amino acid residues with unknown function (Figure 2A). Sequence analysis revealed that the BFA2 protein belongs to the P subfamily and comprises 22 PPR motifs spanning amino acid residues 139–904 (Figure 2A and Supplementary Figure S3). The last PPR motif only contains 32 residues and may represent an incomplete PPR motif (Supplementary Figure S3). Genes with significant sequence identity (more than 50%) to BFA2 are found in eudicotyledons and monocotyledons (Supplementary Figure S4). A blast search also revealed two proteins (PpBFA2-A and PpBFA2-B) in *Physcomitrella patens* (*P. patens*) with low sequence identity to BFA2 (35–38%, Supplementary Figure S4). No genes significantly related to BFA2 were found in *Selaginella moellendorffii* and *Chlamydomonas*. This fact implies that BFA2 may have evolved when land plants including bryophytes originated and was probably lost in the lycophytes during evolution.

BFA2 is predicted to have a putative chloroplast transit peptide of 61 amino acids at its N-terminus. To confirm its chloroplast localization, the DNA region coding for the first 200 amino acids of BFA2 was fused in-frame with GFP in the pBI221 vector and the resulting vector was introduced into Arabidopsis protoplasts by transient transformation. Analysis by confocal laser scanning microscopy showed that the BFA2-GFP signal co-localizes with the chloroplast fluorescence, indicating that BFA2 is targeted to the chloroplast (Figure 2B). To further determine the precise location of BFA2 within chloroplasts, a polyclonal antibody against recombinant BFA2 protein was raised. A signal with a molecular mass of ∼100 kDa (the predicted molecular mass of mature BFA2 is 94 kDa) was detected in the stromal fractions isolated from WT plants, but absent in the stromal fraction from *bfa2* mutants as well as in the thylakoid membranes from WT and *bfa2* plants (Figure 2C). These results indicate that BFA2 is localized to the chloroplast stroma.

### BFA2 Is Required for Accumulation of the *atpH/F* Transcript

**FIGURE 3:**
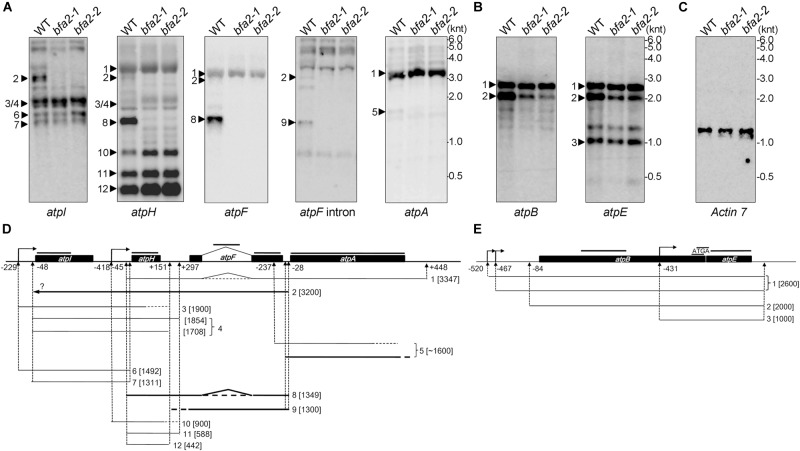
RNA blot analysis of the plastid-encoded ATP synthase genes. **(A–C)** Total RNA isolated from WT and *bfa2* mutants was probed with digoxigenin-labeled probes corresponding to the genes in the large **(A)** and small **(B)** *atp* operons. A blot with the *Actin 7* probe was used as a loading control **(C)**. The positions of RNA markers are shown in kilonucleotides (knt). **(D,E)** Partial transcript map of the *atpI/H/F/A* **(D)** and *atpB/E* **(E)** operons in Arabidopsis. The map was generated based on the transcript size in **(A,B)** as well as on previous reports (Malik Ghulam et al., 2012, 2013). Transcripts absent in *bfa2* are shown as bold black lines. The positions of the probes are indicated above the operons. The numbers refer to the distance upstream of the initiation codon (-) and downstream of the termination codon (+).

Since the PPR proteins are well known to be involved in organelle gene expression, it is very likely that the expression of one or more chloroplast genes encoding ATP synthase subunits is affected in the *bfa2* mutants. To investigate this possibility, we performed RNA gel blot analysis with probes for the large (*atpI/H/F/A*) and the small (*atpB/E*) *atp* operons (Figure 3). For the large *atp* operon, the most striking difference is that the dicistronic *atpH/F* transcript is barely detected in the *bfa2* mutants (Transcript 8; Figure 3A,D), indicating that BFA2 is essential for accumulation of this transcript. However, the level of the monocistronic *atpH* transcripts (transcripts 10, 11, and 12) in the *bfa2* mutants is higher than that in WT (Figure 3A), excluding the possibility that absence of the *atpH/F* transcripts in *bfa2* is due to deficient cleavage between *atpI* and *atpH*. RNA blot analysis using *atpI, atpH, atpF* exon, and *atpF* intron probes also detected a ∼3 kb transcript in WT plants that was absent in the *bfa2* mutants (transcript 2, Figure 3A,D). Given the detection of this transcript with these four probes and its size, it is likely that this transcript corresponds to the unspliced *atpI/H/F* transcript (transcript 2, Figure 3D). The monocistronic unspliced *atpF* transcript was detected with the *atpF* intron probe in the WT plants but was absent in the *bfa2* mutants (transcript 9, Figure 3A).

The *atpA* RNA was mainly detected in the polycistronic *atpH/F/A* transcript (transcript 1, Figure 3A), which is inconsistent with previous reports (Malik Ghulam et al., 2013). In addition to this main polycistronic mRNA, the *atpA* probe also detected a fuzzy weak band around 1600 nucleotides in WT, but the level of this band was significantly reduced in the *bfa2* mutants (transcript 5, Figure 3A,D). As discussed by Malik Ghulam et al. (2013), the monocistronic *atpA* transcript is present in very low amounts *in vivo* and usually possesses truncated 3′ ends whereas most of the 5′ ends of this RNA map at positions -237 (inside the *atpF* mRNA) and -50 (just overlapping with the 3′ end of *atpF*) relative to the *atpA* start codon (Figure 3D; Malik Ghulam et al., 2013). Thus, the weak bands detected in our RNA blot (transcript 5 and several bands below transcript 5) correspond most likely to the monocistronic *atpA* transcript with different 5′ ends, overlapping the *atpF* 3′ end, and truncated 3′ ends (Figure 3D). Reduction of transcript 5 in the *bfa2* mutants indicates that some type of monocistronic *atpA* transcript is unstable in the absence of BFA2.

In the case of the small *atp* operon *atpB/E*, two major bands can be detected by the *atpB* probe (Figure 3B). The monocistronic *atpE* transcript can also be detected by the *atpE* probe (Figure 3B). While the upmost band represents the primary dicistronic *atpB/E* transcript with two isoforms (-520 and -467), the second band corresponds to the processed dicistronic *atpB/E* transcript ending at -84 (Figure 3E, Malik Ghulam et al., 2012). Our results show that the level of the -84 processed *atpB/E* dicistronic mRNA is significantly reduced in the *bfa2* mutants compared with WT plants (Figure 3B,E). The level of primary dicistronic *atpB/E* and the monocistronic *atpE* transcripts are identical in the *bfa2* mutants compared with WT plants (Figure 3E). Reduction of the processed dicistronic *atpB/E* transcript was also observed in the *bfa1-1* and *cgl160* mutants, in which assembly of the chloroplast ATP synthase CF_1_ and CF_o_ subcomplexes, respectively, is less efficient (Rühle et al., 2014; Zhang et al., 2018). Thus, reduction of processed dicistronic *atpB/E* likely represents a secondary effect due to impairment in the assembly of chloroplast ATP synthase.

### Translation Initiation of *atpA* Is Not Affected in the Absence of BFA2

**FIGURE 4:**
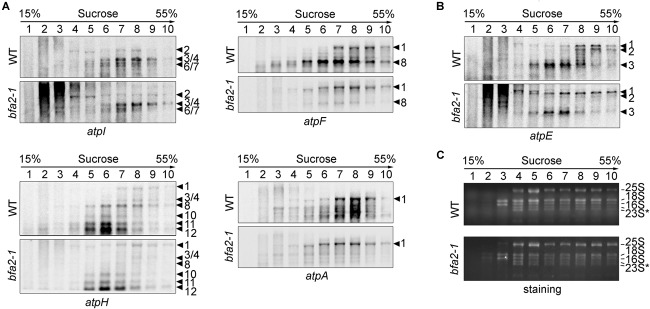
Analysis of polysome association of the plastid transcripts encoding ATP synthase subunits. **(A,B)** Total leaf extracts from wild-type and *bfa2-1* plants were fractionated by centrifugation on 15–55% sucrose density gradients. After centrifugation, the sucrose gradients were divided into 10 fractions of equal volume for RNA isolation. The isolated RNAs were blotted with DIG-labeled DNA probes corresponding to the plastid *atpA, atpF, atpH*, and *atpI* **(A)** as well as *atpE* **(B)** transcripts. The numbers to the right of the panels correspond to the corresponding transcripts illustrated in Figure 3D,E. **(C)** Staining of the rRNA was used as fractionation and loading control. 23S^∗^, two breakdown products of the chloroplast 23S rRNA. The numbers on the right indicate sedimentation coefficients of the major rRNAs.

To rule out the possibility that reduction of the chloroplast ATP synthase in *bfa2* is due to defects in the translation of *atp* mRNAs, we performed a polysome association analysis to investigate translation initiation (Figure 4). Our results show that the distribution of the *atpH/F/A* mRNAs in *bfa2* was slightly shifted toward lower molecular weight fractions compared with WT (transcript 1, Figure 4A). The distribution of other transcripts in the *atpI/H/F/A* operon, such as monomeric *atpH*, was almost identical between *bfa2-1* and WT plants. For the *atpB/E* operon, a clear shift of primary dicistronic *atpB/E* transcript toward lower molecular weight fractions in the *bfa2-1* mutant compared with the wild type was observed (transcript 1, Figure 4B). The shift of the primary dicistronic *atpB/E* transcript is also observed in the *bfa1* and *cgl160* mutants and is unlikely to be the cause for the low accumulation of chloroplast ATP synthase in *bfa2* (Zhang et al., 2018).

**FIGURE 5:**
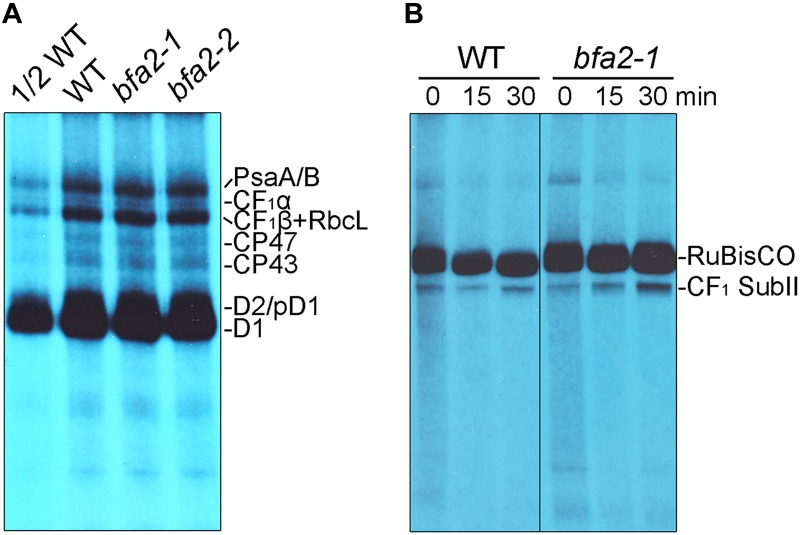
*In vivo* protein labeling analysis of plastid-encoded proteins. **(A)** Pulse labeling of chloroplast thylakoid proteins of WT and *bfa2* mutants. Chloroplast thylakoid proteins were extracted from primary leaves of 12 days old seedlings after labeling with [^35^S]-Met for 20 min, and separated by SDS-urea-PAGE. The labeled proteins were visualized using autoradiography. **(B)** Pulse-chase labeling of chloroplast stromal protein complexes of WT and *bfa2-1* plants. After pulse labeling for 20 min (indicated as 0 min), the leaves were chased with cold Met for 15 and 30 min. Total soluble protein complexes extracted from the leaves were separated by CN-PAGE and the newly assembled complexes were detected by autoradiography.

To investigate whether the alteration of the polysome association with *atpH/F/A* and primary dicistronic *atpB/E* transcripts in *bfa2-1* is responsible for the defect in chloroplast ATP synthase accumulation, *in vivo* protein labeling of the chloroplast proteins with [^35^S]-Met was performed (Figure 5). Cycloheximide, an inhibitor of cytosolic translation, was added to avoid interference with the synthesis of nucleus-encoded proteins. After labeling, thylakoid membranes were isolated and the newly synthesized thylakoid proteins were separated by SDS-PAGE. Radiolabeled thylakoid proteins were detected by autoradiography. The results showed that, as expected, the levels of the newly synthesized PsaA/B, CP47, CP43, D2/pD1, and D1 protein were comparable between WT and *bfa2* mutants (Figure 5A), which is consistent with the fact that *bfa2* is specifically defective in accumulation of chloroplast ATP synthase. For the chloroplast ATP synthase CF_1_α subunit, a very weak signal was detected below the PsaA/B subunits and its level is identical in both WT and two *bfa2* mutant genotypes (Figure 5A). The levels of newly synthesized CF_1_β subunits of as well as RbcL contamination in thylakoids were also identical in *bfa2* and WT plants after labeling for 20 min (Figure 5A).

CF_1_α and CF_1_β are components of the ATP synthase CF_1_ subcomplex. To further prove that protein synthesis of CF_1_α and CF_1_β and their subsequent incorporation into functional CF_1_ is not affected in *bfa2*, we analyzed the assembly of the CF_1_ subcomplex in the chloroplast stroma by pulse-chase labeling. We designated this subcomplex CF_1_ SubII in our previous report (Figure 5B, Zhang et al., 2018), and it contains subunits of CF_1_α, CF_1_β, CF_1_γ, CF_1_ε, and CF_1_δ, but not CF_o_I, which is the product of *atpF* (Zhang et al., 2016). Our results show that formation of CF_1_ SubII is as efficient in *bfa2-1* as in WT plants after pulse-labeling for 20 min and a subsequent chase for 15 and 30 min (Figure 5B). These results are different from those obtained with *bfa1* and *bfa3* (Zhang et al., 2016, 2018), further confirming that synthesis of CF_1_α and CF_1_β is not affected in *bfa2*, although the level of processed dicistronic *atpB/E* was reduced and polysome association with *atpH/F/A* and primary dicistronic *atpB/E* was slightly altered in the *bfa2* mutants (Figure 3, 4).

Taken together, we conclude that absence of the dicistronic *atpH/F* is the primary cause for the decreased accumulation of chloroplast ATP synthase in *bfa2*, and that BFA2 is likely directly required for the accumulation of the RNAs with a 3′-end or 5′-end mapping between *atpF* and *atpA* (Figure 3A,D). The BFA2 protein belongs to the P class PPR proteins and this class of proteins can act as barrier to prevent the RNA degradation by exoribonucleases (Barkan and Small, 2014). Since PPR10 binds to the 5′ termini of *atpH/F*, we hypothesize that BFA2 binds to the 3′ termini of *atpH/F* as well as to other transcripts overlapping the intergenic region of *atpF-atpA* (Figure 3D).

### BFA2 Binds to the Consensus Sequence in the *atpF* 3′-UTR and *atpA* 5′-UTR

**FIGURE 6:**
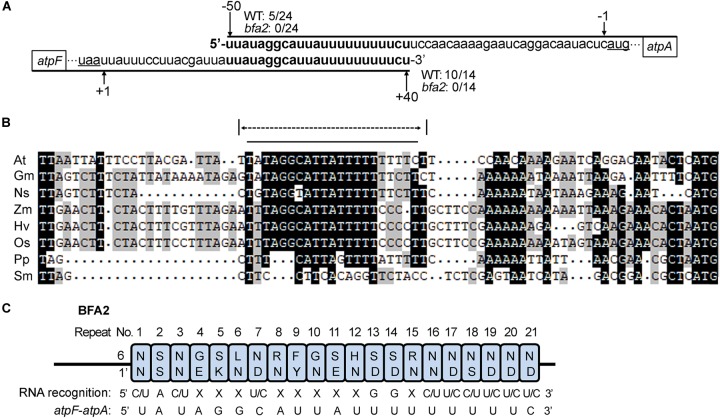
Position of the BFA2-dependent RNA termini. **(A)** Overlapping transcript termini in the *atpF/A* intergenic region. Nucleotide positions relative to the start codon of *atpA* (-50 and -1) and stop codon of *atpF* (+40 and +1) are shown above and below the sequences, respectively. The numbers of 5/24, 0/24, 10/14, and 0/14 represent the ratio of the clones with a 5′ or 3′ end positions from the WT or *bfa2* samples in the cRT-PCR analyses (Supplementary Figure S5 and Supplementary Table S1). **(B)** Alignment of the *atpF/A* intergenic regions (from the stop codon of *atpF* to the start codon of *atpA*). The overlapping transcript termini in the *atpF/A* intergenic regions and the sequence predicted for BFA2 binding are indicated by the dotted and black lines, respectively. Nucleotides U are indicated by T. At, *Arabidopsis thaliana*; Gm, *Glycine max*; Os, *Oryza sativa*; Zm, *Zea mays*; Ns, *Nicotiana sylvestris*; Hv, *Hordeum vulgare*; Pp, *Physcomitrella patens*; Sm, *Selaginella moellendorffii*. **(C)** Prediction of the nucleotide-binding site for BFA2. The residues that determine nucleotide-binding specificity (residues 6 and 1’ in Supplementary Figure S3) in each PPR motif were extracted according to Barkan et al. (2012). The overlapping sequence in the *atpF/A* intergenic RNA (from the second nucleotide) is shown.

A small RNA (sRNA) corresponding to the *atpF* 3′ region was detected in barley and rice and this sRNA is predicted to be the footprint of uncharacterized PPR-like proteins (Ruwe and Schmitz-Linneweber, 2012; Zhelyazkova et al., 2012). These facts led us to propose that the sRNA from the *atpF-atpA* intergenic region is the footprint of BFA2 in Arabidopsis. To confirm this hypothesis, we first determined the transcript termini in the *atpF-atpA* intergenic region by circularization RT-PCR (cRT-PCR) (Supplementary Figure S5). Consistent with the results reported by Malik Ghulam et al. (2013), our results show that most clones (10 out of 14) had their 3′ end at position +40 from the *atpF* stop codon and 5 out of 24 clones had their 5′ end at position -50 from the *atpA* start codon in WT (Figure 6A and Supplementary Table S1). However, neither 3′ ends of *atpF* nor 5′ ends of *atpA* were mapped to these two positions in *bfa2* (Figure 6A and Supplementary Table S1). These results suggest that BFA2 binds to the *atpF-atpA* intergenic region to stabilize the corresponding mRNA *in vivo*. Moreover, the overlapping region comprises 23 residues and is basically consistent with the number of PPR motifs (22 PPR motifs) of the BFA2 protein (Figure 6A).

Alignment analysis showed that the overlapping region (except for the first residue) in the *atpF* 3′-UTR and *atpA* 5′-UTR in Arabidopsis is highly conserved in Angiosperms, but not in *Physcomitrella patens* and *Selaginella moellendorffii* (Figure 6B), which is in line with the fact that two proteins (PpBFA2-A and PpBFA2-B) in *Physcomitrella patens* (*P. patens*) show low sequence identity with BFA2 (35–38%) and that no BFA2-like protein was found in *S. moellendorffii* (Supplementary Figure S4). Although the last 6 residues vary among different plant species, most residues in this region are U and C (Figure 6B). Since the first residue is not conserved (Figure 6B), BFA2 may bind to the 22 conserved residues from the second residue in the sRNA. To confirm our hypothesis, the potential binding sequence of BFA2 was predicted according to the PPR code established previously (Barkan et al., 2012). As shown in Figure 6C, the 21 nucleotides predicted to bind by the 21 PPR motifs of BFA2 are (C/U)A(C/U)XXX(U/C)XXXXXGGX(C/U)(U/C)(C/U)(U/C)(U/C)(U/C). While X represents any nucleotide that cannot be precisely predicted, the nucleotides in parentheses are optional. Among the 21 nucleotides, 10 of them match with the corresponding residues in the overlapping transcript termini of the *atpF-atpA* intergenic region (Figure 6C). For the 5th and 11th PPR motifs, serine (S) was identified at position 6 (Figure 6C). It has been suggested that S_6_ shows a strong correlation with purines (Barkan et al., 2012), which is consistent with fact that G and A were found in the corresponding position of the *atpF-atpA* intergenic region (Figure 6C). These results support our view that BFA2 binds to the overlapping transcript termini in the *atpH-atpA* intergenic region starting from the second residue.

**FIGURE 7:**
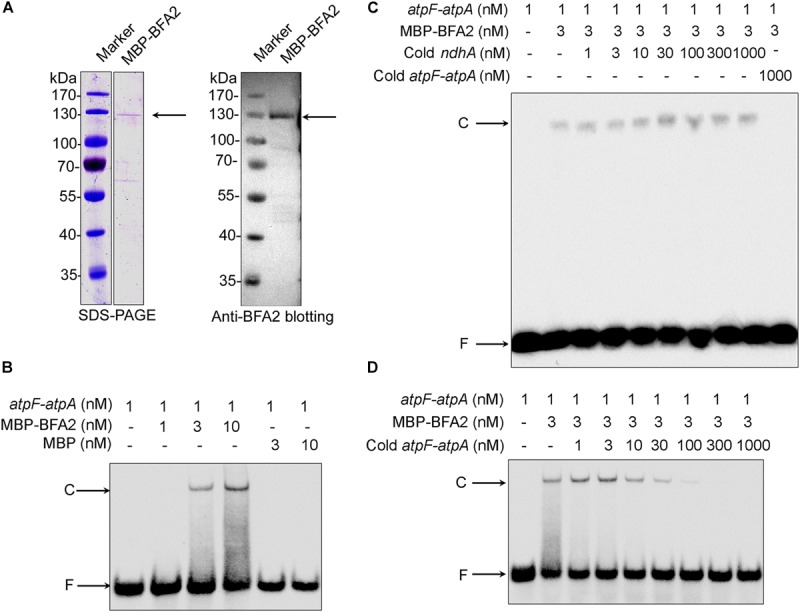
Gel mobility shift assays of the RNA binding activity of BFA2. **(A)** Purified MBP-BFA2 used in the RNA binding assays. The purified recombinant BFA2 was separated by SDS-PAGE and visualized by Coomassie Brilliant Blue (CBB) staining (left) and immunoblotting with anti-BFA2 antibody (right). **(B)** Gel mobility shift assays demonstrating that MBP-BFA2 binds with high affinity to the *atpF* sRNA. The concentrations of labeled sRNA probe, recombinant BFA2, and recombinant MBP are indicated above each lane. C and F indicate the BFA2-RNA complex and free labeled RNA, respectively. **(C,D)** Competition experiments using nonlabeled *ndhA* sRNA **(C)** and nonlabeled *atpF-atpA* sRNA **(D)**. The concentrations of probes and protein are indicated above the gel. In each reaction, 1 μg yeast tRNA was added to lower the background of nonspecific RNA binding.

*In vitro* electrophoretic mobility shift assays (EMSA) were performed. Recombinant mature BFA2 protein fused with the MBP (maltose-binding protein) tag was expressed in *Escherichia coli* (*E. coli*) and purified (Figure 7A). The molecular mass of the purified fusion protein is about 130 kDa and is consistent with the predicted molecular mass of BFA2-MBP (136 kDa). The biotinylated RNA corresponding to the overlapping transcript termini in *atpF-atpA* was chemically synthesized and incubated with the BFA2-MBP fusion protein. The BFA2-RNA complex can be detected when the protein molar concentration is three times higher than that of the RNA (Figure 7B). There was no shift when the biotinylated RNA was incubated with MBP protein (Figure 7B). A set of competition assays were performed to confirm the binding specificity of BFA2. The 5′ end of *ndhA* mRNA has been shown to be the binding site of PGR3 (PROTON GRADIENT REGULATION 3) (Cai et al., 2011). Even addition of 1000-fold amount of cold *ndhA* mRNA did not affect the formation of the BFA2-RNA complex (Figure 7C). However, the addition of >30-fold amount of unlabeled *atpF-atpA* RNA significantly inhibited the binding of BFA2 with the labeled RNA probe (Figure 7D). These results clearly demonstrate that BFA2 protein binds to the *atpF-atpA* intergenic region in a sequence-specific manner.

## Discussion

The plastid-encoded *atpF* gene encodes the CF_o_I subunit of the chloroplast ATP synthase. CF_o_I interacts with the *atpG* product CF_o_II to form the peripheral stalk holding CF_o_ and CF_1_ together (Rühle and Leister, 2015). In chloroplasts, the *atpF* RNA is solely detected in the polycistronic *atpH/F/A* and dicistronic *atpH/F* transcripts (Figure 3A; Malik Ghulam et al., 2013). Analysis of chloroplast small RNAs (sRNAs) in rice and barely reveals two sRNAs mapping at the two ends of dicistronic *atpH/F* mRNA, respectively (Ruwe and Schmitz-Linneweber, 2012; Zhelyazkova et al., 2012). Both of them are predicted to represent footprints of PPR proteins (Zhelyazkova et al., 2012; Malik Ghulam et al., 2013). While the sRNA at the 5′-end of *atpH/F* includes the binding site for PPR10 (Pfalz et al., 2009; Prikryl et al., 2011), the putative PPR protein binding to the 3′-end of the dicistronic *atpH/F* transcript was not yet known. In this study, we provide evidence that P-class PPR protein BFA2 binds to this site.

Our conclusion is mainly supported by the following evidence. (1) The level of the chloroplast ATP synthase is specifically reduced in the absence of BFA2, while accumulation of other thylakoid complexes is not affected (Figure 1E and Supplementary Figure S2). This is also consistent with the photosynthetic properties detected in *bfa2* (Figure 1 and Supplementary Figure S1). (2) Dicistronic *atpH/F* transcript is absent in *bfa2* and other transcripts with termini in the intergenic region of *atpF-atpA* also appear to be unstable in the absence of BFA2 (Figure 3). (3) The BFA2 binding site was predicted to cover the overlapping region between the 3′ end of *atpF* and the 5′ end of *atpA* (Figure 6). (4) EMSA analyses showed that BFA2 protein binds to the *atpF-atpA* intergenic region in a sequence-specific manner (Figure 7). Sequence alignment analysis showed that BFA2 belongs to the P-class PPR proteins with 22 PPR motifs (Supplementary Figure S3). Our results suggest that BFA2 acts analogously to other typical PPR proteins such as PPR10, PGR3, and HCF152, by directly binding to the *atpF*-*atpA* intergenic region to prevent degradation of BFA2-dependent transcripts by blocking exoribonucleases acting either from the 5′ or 3′ ends (Barkan and Small, 2014). However, because several nucleotides that bind to the PPR motifs in BFA2 can not be precisely predicted (Figure 6C), BFA2 may have another binding site(s) in the chloroplast-encoded genes, which need to be investigated in the further analyses.

For some P-class PPR proteins like PPR10, they not only act as site-specific barriers to protect target RNA segments by blocking exoribonuclease intruding from either direction, but also remodel the structure of ribosome-binding sites in the target RNA to enhance translation (Prikryl et al., 2011). Since BFA2 binds to the intergenic regions of *atpF-atpA*, which is just upstream of the start codon of *atpA*, is it possible that binding of BFA2 in this region releases the ribosome binding site of *atpA*? In Arabidopsis, monomeric *atpA* transcript was barely detectable in chloroplasts (Malik Ghulam et al., 2013; Figure 3). Thus, *atpA* translation should arise from the polycistronic *atpH/F/A* transcript. Although polysome association with *atpH/F/A* transcript was slightly reduced in the *bfa2* mutant (Figure 4A), CF_1_α synthesis and subsequent assembly into CF_1_ were not affected (Figure 5). These facts suggest that binding of BFA2 in the intergenic region of *atpF-atpA* is not required for the translation of *atpA*. However, we cannot fully rule out the possibility that BFA2 is involved in the activation of *atpA* translation since no solid evidence was obtained by more direct approaches like polysome profiling.

Our results demonstrate that absence of dicistronic *atpH/F* transcript is the main cause of the low ATP synthase accumulation phenotype of *bfa2* (Figure 3–5). The dicistronic *atpH/F* transcript is barely detectable in *bfa2* (Figure 3). This raises the question of how the *atpF* product CF_o_I can accumulate to about one-quarter in *bfa2* as compared to WT (Figure 1)? One possibility is that *atpF* translation proceeds to a small extent from the polycistronic *atpH/F/A* transcript which accumulates normally in the *bfa2* mutants (Figure 3).

Homologs of BFA2 are found in angiosperms, consistent with the highly conserved intergenic regions of *atpF-atpA* among angiosperms (Figure 6B and Supplementary Figure S4, Zhelyazkova et al., 2012). Moreover, two putative BFA2 homologs were found in *P. patens* although they display low sequence identity with BFA2 from higher plants (Supplementary Figure S4). However, although a ∼20 nt sequence in the *atpF-atpA* regions from *P. patens* chloroplasts shows high similarity to the BFA2-binding sequence of higher plants, a 3 nt deletion was found in this sequence (Figure 6B). Moreover, this sequence is located just downstream of the stop codon of *atpF* (Figure 6B). It is reasonable to assume that translation termination may be affected if the BFA2-like proteins in *P. patens* bind to this region. Thus, detailed analyses are necessary to clarify the function of these two proteins in *P. patens*.

In summary, our genetic approaches have identified a P-class PPR protein BFA2, which is specifically required for the normal accumulation of chloroplast ATP synthase. We have demonstrated that BFA2 binds to the intergenic region of *atpF-atpA* and mainly acts as a site-specific barrier to protect *atpH/F* mRNA by blocking exoribonuclease degradation from the 3′-direction. Thus, stabilization of the *atpH/F* transcript requires two independent PPR proteins, PPR10 and BFA2, to protect the mRNA against exoribonucleases.

## Significance Statement

In this study, we discovered a chloroplast PPR protein BFA2, which protects target mRNAs from degradation by exoribonucleases by binding to the consensus sequence of the *atpF-atpA* intergenic region.

## Author Contributions

LZ, WL, and LP conceived the study and designed the experiments. LZ, WL, WZ, and LC performed the experiments. LZ and WL produced the figures. LZ, WL, J-DR, and LP wrote the manuscript. LP supervised the whole study. All authors analyzed the data.

## Conflict of Interest Statement

The authors declare that the research was conducted in the absence of any commercial or financial relationships that could be construed as a potential conflict of interest.
