# Supplementary material for: PPR Protein BFA2 Is Essential for the Accumulation of the atpH/F Transcript in Chloroplasts
Source: Front Plant Sci. 2019 Apr 12;10:446. doi: 10.3389/fpls.2019.00446 (PMC6474325; doi:10.3389/fpls.2019.00446)
Supplement: Supplementary file 1 [file Data_Sheet_1.docx]

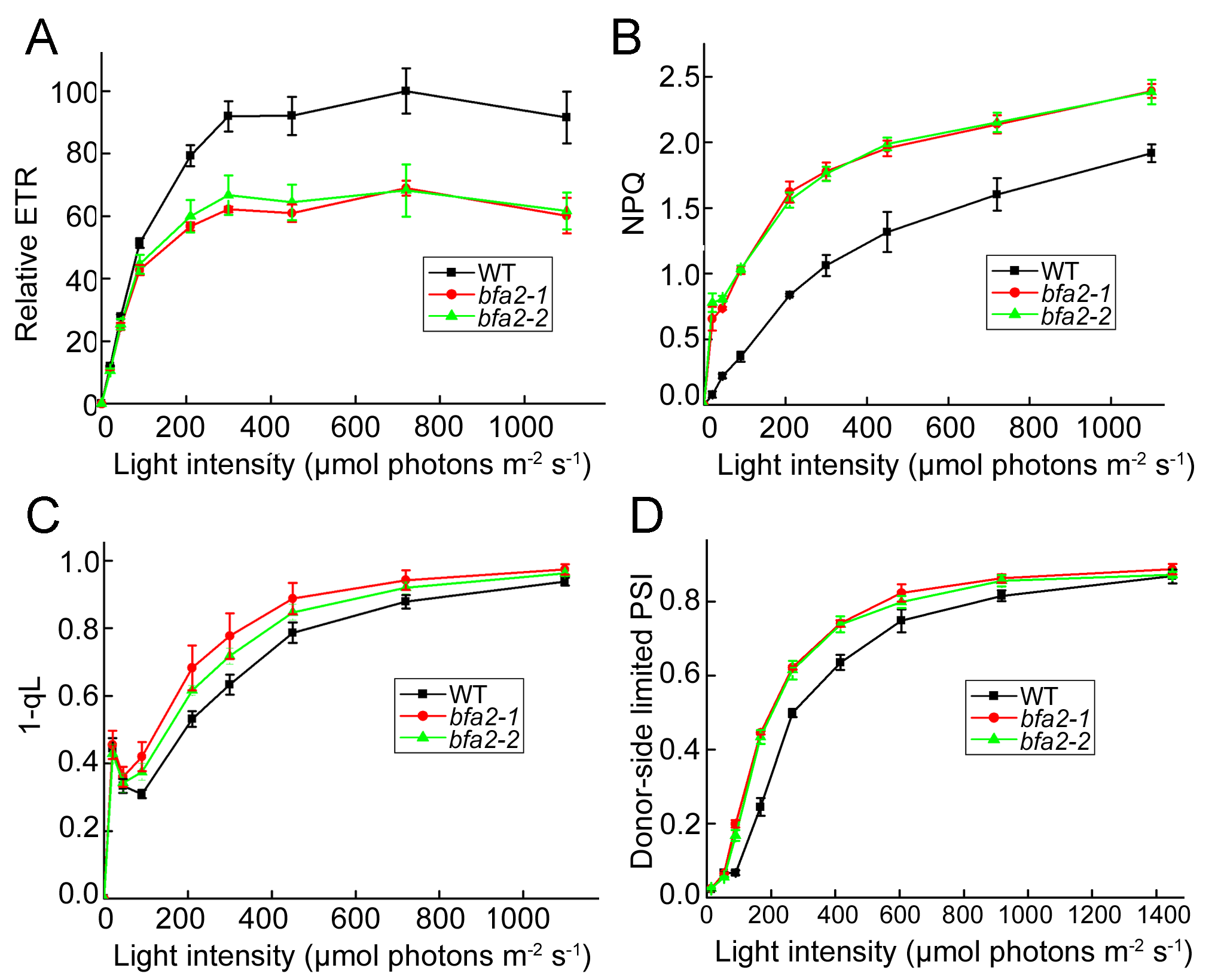


**FIGURE S1. Photosynthetic properties of *bfa2*.** Light intensity dependence of ETR **(A)**, NPQ **(B)**, 1-qL **(C)**, and oxidation of the donor side of PSI **(D)**. Means ± SD (n = 4).


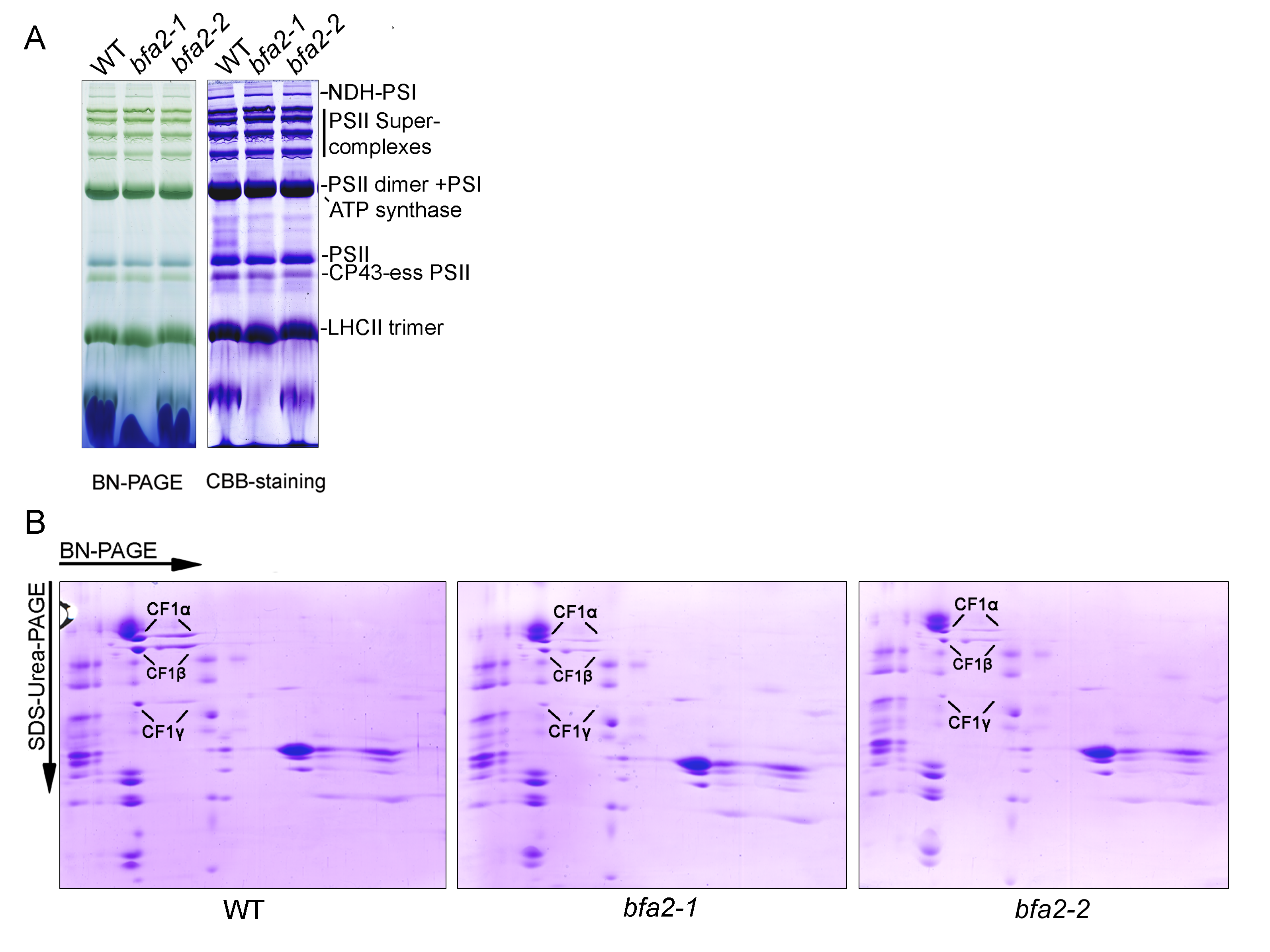


**FIGURE S2. Accumulation of thylakoid protein complexes in *bfa2*.** **(A)** BN-PAGE analysis of the thylakoid protein complexes in *bfa2* and WT plants. BN-gels were stained with Coomassie Brilliant Blue (CBB). NDH-PSI, NDH-PSI supercomplex. **(B)** 2D BN/SDS-Urea-PAGE separation of thylakoid protein complexes in *bfa2* and WT plants. Thylakoid protein complexes were separated by BN-PAGE **(A)** and the proteins were further resolved by SDS-urea-PAGE. The gels were stained with CBB and the spots corresponding to the CF_1_α, CF_1_β, and CF_1_γ subunits are indicated.


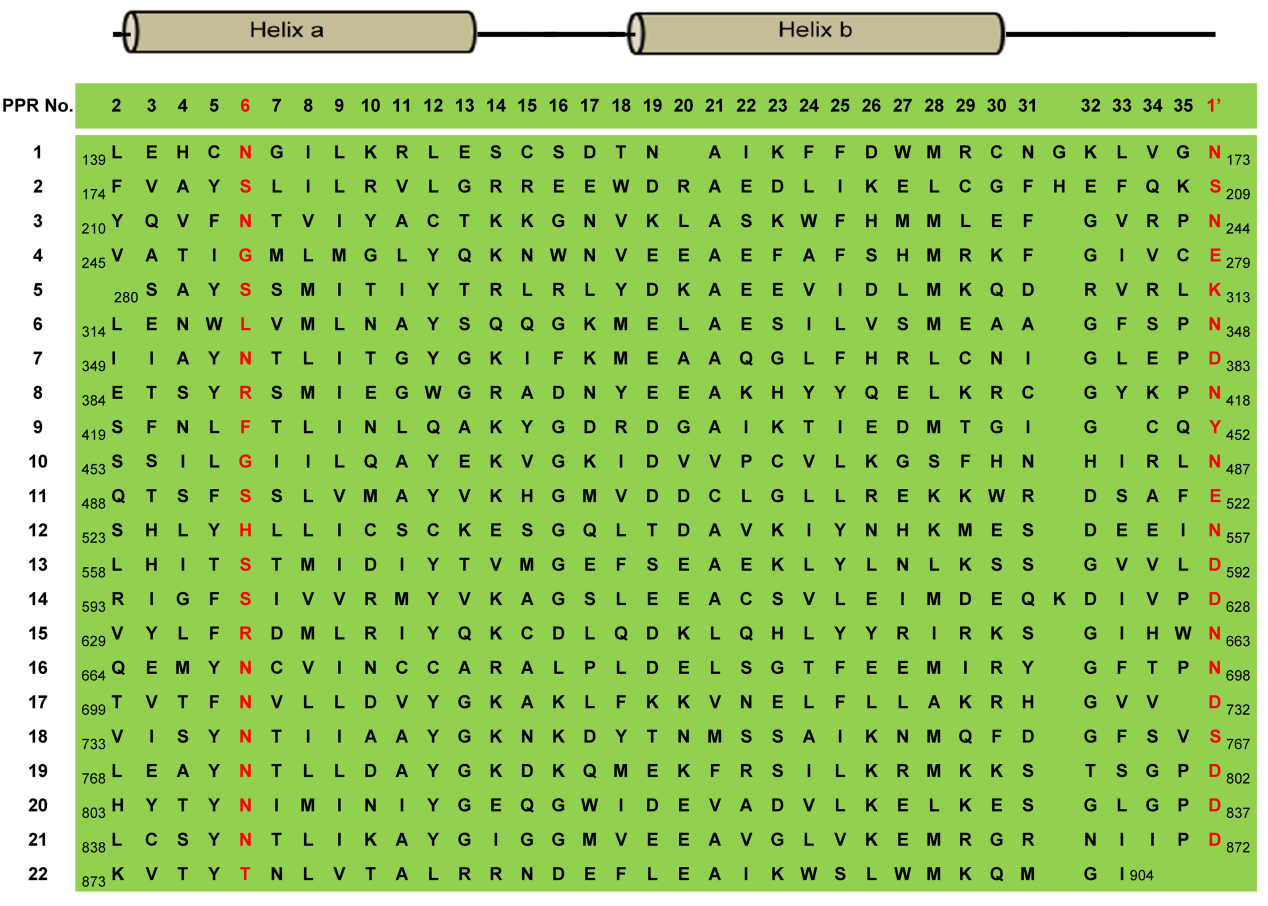


**FIGURE S3. Sequence alignment of the 22 PPR domains in BFA2.** The residues required for RNA-binding specificity at the 6th and 1’th positions in each PPR motif are highlighted in red. The numbering system of PPR motifs is taken from Barkan et al. (2012).


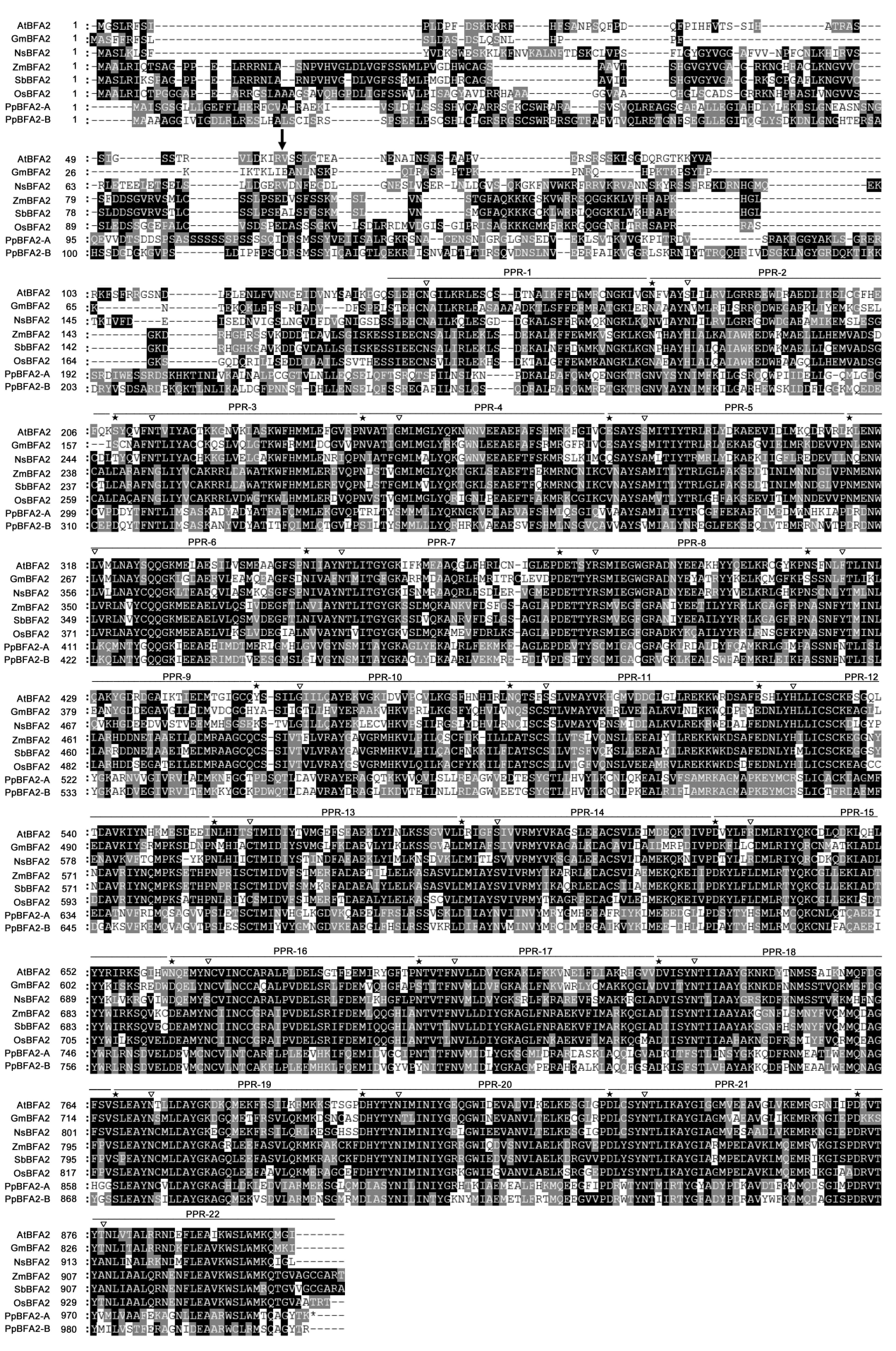


**FIGURE S4. Alignment of BFA2 homologs.** The alignment was performed with Genedoc. Identical and conserved amino acids are shaded in black and grey, respectively. The predicted cleavage site of AtBFA2 is indicated by an arrowhead. The positions of the 22 PPRs are indicated by the black lines above the sequences. In each PPR motif, the 6th and 1’th residues required for RNA-binding specificity are indicated by triangles and asterisks, respectively. AtBFA2 (AT4G30825, *Arabidopsis thaliana*), GmBFA2 (Glyma.04G155800, *Glycine max*), OsBFA2 (Os09g25550, *Oryza sativa*), ZmBFA2 (XP_008662784, *Zea mays*), NsBFA2 (XP_009792607.1, *Nicotiana sylvestris*), SbBFA2 (SORBIDRAFT_07g007540, *Sorghum bicolor*), PpBFA2-A (Pp3c16_4140, *Physcomitrella patens*), PpBFA2-B (Pp3c5_2530, *Physcomitrella patens*).

**
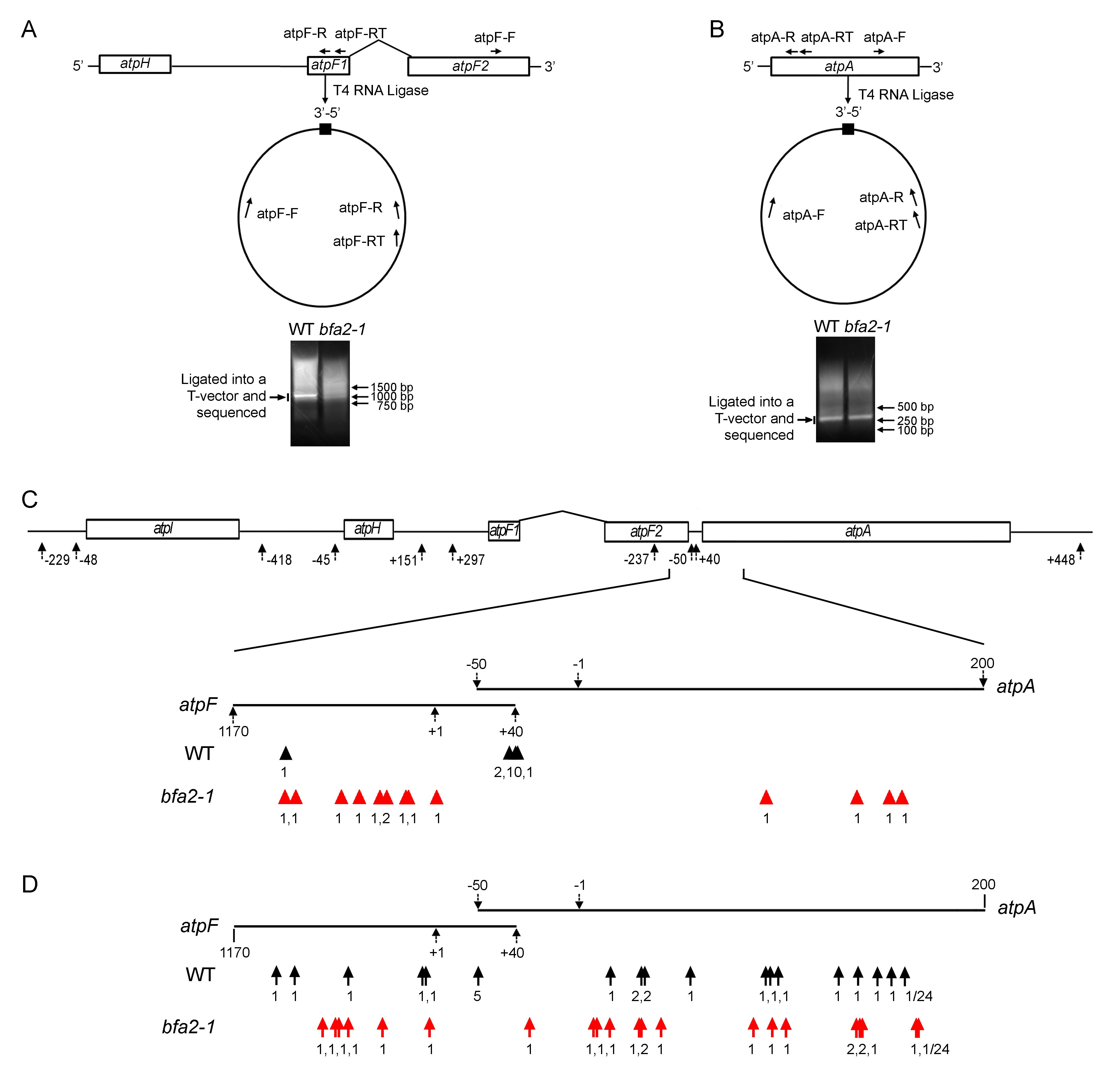
FIGURE S5. Mapping of *atpF* and *atpA* transcript termini. (A)** cRT-PCR procedure used to map the ends of the *atpF* transcript. RNA isolated from wild-type and *bfa2-1* was circularized by ligation and the atpF-RT primer was used for reverse transcription. PCR was performed using atpF-F and atpF-R primers, whose sequences are listed in **Table S2**. The PCR product indicated by a line was ligated into a T-vector for sequencing. **(B)** cRT-PCR procedure used to map the ends of the *atpA* transcript. Primer atpA-RT was used for reverse transcription. Primers of atpA-F and atpA-R were used for PCR **(Table S2)**. The PCR product indicated by a line was ligated into a T-vector for sequencing. **(C)** Schematic showing of the position of the 3’ end of *atpF* transcripts by cRT-PCR analysis. The schematic presentation of the *atpI/H/F/A* operon is shown in the top panel and the region including the 3’ end of *atpF* and 5’ end of the *atpA* transcripts ends is indicated in the bottom panel. The numbers with dotted arrowheads refer to the distance upstream of the initiation codon (-) and downstream of the termination codon (+), respectively. Black and red triangles present the position of 3’ end of *atpF* transcripts from WT and *bfa2-1*, respectively. The detailed positions are listed in Table S1. The numbers below triangles correspond to the clone number displaying a specific 3’ transcript end. **(D)** Schematic presentation of the 5’ end of the *atpA* transcripts by cRT-PCR analysis as in **(C)**. Black and red arrowheads present the position of 5’ end of transcripts from WT and *bfa2-1*, respectively. The detailed positions are listed in Table S1. The numbers below arrowheads correspond to the clone number displaying a specific 5’ transcript end.

**TABLE S1.** Summary of the cRT-PCR analysis of the *atpF* and *atpA* transcripts.

| Summary of cRT-PCR analysis of the *atpF* transcripts | | | | |  |
| --- | --- | --- | --- | --- | --- |
| clone ID | | Position of 5' end relative to *atpF* start codon | Position of 3' end relative to *atpF* start codon | Sequence at the ligated 3'-5' junction |  |
| WT | 49 | -34 | +40 | …ATTATTTTTTTTTCT-3'/5'-CCTTTTTTATAGTTT… |  |
|  | 50 | -400 | +40 | …ATTATTTTTTTTTCT-3'/5'-CCTACAATTATTCAT… |  |
|  | 51 | -12 | 1196 | …TAAATAGTTGTTTGA-3'/5'-AGAGGAGATTATATG… |  |
|  | 55 | -48 | +40 | …ATTATTTTTTTTTCT-3'/5'-AAAAGGACAGAGTTC… |  |
|  | 57 | -166 | +40 | …ATTATTTTTTTTTCT-3'/5'-TTTGGAAAATACGTT… |  |
|  | 58 | 30 | +40 | …ATTATTTTTTTTTCT-3'/5'-CTTGGGTCACTGGCC… |  |
|  | 60 | -32 | +40 | …ATTATTTTTTTTTCT-3'/5'-TTTTTTATAGTTTAG… |  |
|  | 63 | -3 | +40 | …ATTATTTTTTTTTCT-3'/5'-ATATGAAAAATTTAA… |  |
|  | 64 | -58 | +37 | …GGCATTATTTTTTTT-3'/5'-GAATAAAAAAAAAAG… |  |
|  | 67 | -272 | +37 | …GGCATTATTTTTTTT-3'/5'-GTTGCGAAAAAAGTA… |  |
|  | 68 | -61 | +40 | …ATTATTTTTTTTTCT-3'/5'-AAAGAATAAAAAAAA… |  |
|  | 70 | -475 | +40 | …ATTATTTTTTTTTCT-3'/5'-CCTTTTGTTTAAGCC… |  |
|  | 71 | -382 | +40 | …ATTATTTTTTTTTCT-3'/5'-GACAACAATCCTTGG… |  |
|  | 72 | 2 | +41 | …TTATTTTTTTTTCTT-3'/5'-TGAAAAATTTAACCG… |  |
| *bfa2-1* | 73 | -139 | 1233 | …TATTAATGCAAATAT-3'/5'-AGGTTTTTGATTCTG… |  |
|  | 78 | -56 | +163 | …AATACCGGTACCGTA-3'/5'-ATAAAAAAAAAAGGA… |  |
|  | 79 | 10 | 1243 | …AATATTGGGATGTTT-3'/5'-TTAACCGATTCTTTC… |  |
|  | 80 | -93 | 1255 | …TTTGGTACGATGAAA-3'/5'-ATTAAATTCAAATTT… |  |
|  | 82 | 14 | +232 | …GGTGAATTAGTAGAA-3'/5'-CCGATTCTTTCGTTT… |  |
|  | 84 | -121 | 1196 | …TAAATAGTTGTTTGA-3'/5'-ACAAATATCCAAATA… |  |
|  | 85 | -146 | 1247 | …TTGGGATGTTTGGTA-3'/5'-ATAGAATAGGTTTTT… |  |
|  | 86 | -2 | 1201 | …AGTTGTTTGAGTAAT-3'/5'-ATATGAAAAATTTAA… |  |
|  | 87 | -46 | +226 | …ATGGCAGGTGAATTA-3'/5'-AAGGACAGAGTTCCT… |  |
|  | 90 | -139 | 1256 | …TTGGTACGATGAAAG-3'/5'-AGGTTTTTGATTCTG… |  |
|  | 91 | -70 | 1247 | …TTGGGATGTTTGGTA-3'/5'-TTTCAATAAAAAGAA… |  |
|  | 92 | -106 | 1270 | …GAAATAACTGATTAA-3'/5'-GGTAAATTAAATTAT… |  |
|  | 95 | -173 | 1225 | …TTACGTACTATTAAT-3'/5'-TAAAAATTTTGGAAA… |  |
|  | 96 | -138 | +207 | …TTATGGTCTTGATGA-3'/5'-GGTTTTTGATTCTGT… |  |
| Summary of cRT-PCR analysis of the *atpA* transcripts | | | | | |
| clone ID | | Position of 5' end relative to atpA start codon | Position of 3' end relative to atpA start codon | Sequence at the ligated 3'-5' junction |  |
| WT | 1-1 | -50 | 1181 | …ATTCGCTGAATTAGA-3'/5'-TTATAGGCATTATTT… |  |
|  | 1-3 | -141 | 1162 | …TAAAATTGGAATTGG-3'/5'-GTAATGAGTTACATT… |  |
|  | 1-6 | 101 | 1397 | …ACGCACTTACTTAAA-3'/5'-AAGTGGGCGATGGCA… |  |
|  | 1-8 | 35 | 1169 | …TGGAATTGGCTCAAT-3'/5'-TTATCCGTGAACGTA… |  |
|  | 1-9 | 159 | 1169 | …GGAATTGGCTCAATT-3'/5'-AGTAATGGCAGGTGA… |  |
|  | 1-10 | -50 | 1181 | …ATTCGCTGAATTAGA-3'/5'-TTATAGGCATTATTT… |  |
|  | 1-11 | -114 | 1160 | …ATTAAAATTGGAATT-3'/5'-ATGCAAATATTGGGA… |  |
|  | 1-13 | -149 | 1168 | …TGGAATTGGCTCAAT-3'/5'-TTGTTTGAGTAATGA… |  |
|  | 1-14 | -76 | 1169 | …GGAATTGGCTCAATT-3'/5'-ACTGATTAATTATTT… |  |
|  | 1-15 | -74 | 1181 | …ATTCGCTGAATTAGA-3'/5'-TGATTAATTATTTCC… |  |
|  | 1-22 | 16 | 1169 | …GGAATTGGCTCAATT-3'/5'-GCCGACGAAATTAGT… |  |
|  | 2-2 | -50 | 1181 | …ATTCGCTGAATTAGA-3'/5'-TTATAGGCATTATTT… |  |
|  | 2-3 | 156 | 1361 | …AGAAATTGGACAAGT-3'/5'-ATTAGTAGAATTTGA… |  |
|  | 2-6 | 58 | 1231 | …CTACTCAGAATCAAT-3'/5'-TATAATAGAGAAGTA… |  |
|  | 2-10 | 97 | 1169 | …GGAATTGGCTCAATT-3'/5'-CTTCAAGTGGGCGAT… |  |
|  | 2-12 | 95 | 1181 | …ATTCGCTGAATTAGA-3'/5'-TACTTCAAGTGGGCG… |  |
|  | 2-13 | 162 | 1484 | …GAAAGAAGGTATTCA-3'/5'-AGAATTTGAGGAGGG… |  |
|  | 2-14 | 140 | 1166 | …ATTGGAATTGGCTCA-3'/5'-AAGTAATGGCAGGTG… |  |
|  | 2-15 | 34 | 1175 | …GGCTCAATTCGCTGA-3'/5'-ATTATCTGTGAACGT… |  |
|  | 2-16 | -50 | 1181 | …ATTCGCTGAATTAGA-3'/5'-TTATAGGCATTATTT… |  |
|  | 2-21 | 34 | 1166 | …ATTGGAATTGGCTCA-3'/5'-ATTATCCGTGATCGT… |  |
|  | 2-22 | 131 | 1181 | …ATTCGCTGAATTAGA-3'/5'-GTCTTGATGAAGTAA… |  |
|  | 2-23 | 35 | 1169 | …GGAATTGGCTCAATT-3'/5'-TTATCCGTGAACGTA… |  |
|  | 2-24 | -50 | 1179 | …CAATTCGCTGAATTA-3'/5'-TTATAGGCATTATTT… |  |
| *bfa2-1* | 1-25 | -118 | 1179 | …GGCCCAATTCGCTGA-3'/5'-ATTAATGCAAATATT… |  |
|  | 1-29 | -96 | 1181 | …ATTCGCTGAATTAGA-3'/5'-TTGGTACGATGAAAG… |  |
|  | 1-31 | -114 | 1181 | …ATTCGCTGAATTAGA-3'/5'-ATGCAAATATTGGGA… |  |
|  | 1-33 | 26 | 1153 | …CTGGAAAATTAAAAT-3'/5'-TTAGTAATATTATCC… |  |
|  | 1-35 | 89 | 1196 | …AGCCTTTTCCCAATT-3'/5'-GTACCGTACTCCAAG… |  |
|  | 1-36 | 169 | 1181 | …ATTCGCTGAATTAGA-3'/5'-GAGGAGGGTACTATA… |  |
|  | 1-38 | 168 | 1278 | …AAACAATCCCAATCA-3'/5'-TGAGGAGGGTACTAT… |  |
|  | 1-39 | 34 | 1175 | …GGCTCAATTCGCTGA-3'/5'-ATTATCCGTGAACGT… |  |
|  | 1-40 | -23 | 1169 | …GGAATTGGCTCAATT-3'/5'-ACAAAAGAATCAGGA… |  |
|  | 1-41 | 141 | 1250 | …AAGAGGTCAACGATT-3'/5'-AGTAATGGCAGGTGA… |  |
|  | 1-42 | 33 | 1177 | …CTCAATTCGCTGAAT-3'/5'-TATCATCCGTGAACG… |  |
|  | 2-50 | -127 | 1252 | …GAGGTCAACGATTGC-3'/5'-TTACGTACTATTAAT… |  |
|  | 2-51 | 98 | 1169 | …GGAATTGGCTCAATT-3'/5'-TTCAAGTGGGCGATG… |  |
|  | 2-55 | 139 | 1179 | …CAATTCGCTGAATTA-3'/5'-GAAGTAATGGCAGGT… |  |
|  | 2-56 | 34 | 1180 | …AATTCGCTGAATTAG-3'/5'-ATTATCCGTGAACGT… |  |
|  | 2-60 | 11 | 1168 | …TGGAATTGGCTCAAT-3'/5'-TTAGAGCCGACGAAA… |  |
|  | 2-61 | -72 | 1166 | …ATTGGAATTGGCTCA-3'/5'-ATTAATTATTTCCTT… |  |
|  | 2-63 | 139 | 1179 | …CAATTCGCTGAATTA-3'/5'-GAAGTAATGGCAGGT… |  |
|  | 2-65 | 104 | 1169 | …GGAATTGGCTCAATT-3'/5'-TGGGCGATGGCATCG… |  |
|  | 2-66 | 44 | 1181 | …ATTCGCTGAATTAGA-3'/5'-AACGTATTGAGCAAT… |  |
|  | 2-70 | 141 | 1169 | …GGAATTGGCTCAATT-3'/5'-AGTAATGGCAGGTGA… |  |
|  | 2-71 | 142 | 1170 | …GAATTGGCTCAATTC-3'/5'-GTAATGGCAGGTGAA… |  |
|  | 2-72 | -120 | 1197 | …GCCTTTTCCCAATTT-3'/5'-CTATTAATGCAAATA… |  |
|  | 2-73 | 8 | 1169 | …GGAATTGGCTCAATT-3'/5'-CCATTAGAGCCGACG… |  |

Clones with their 3’ end at position +40 from the *atpF* stop codon and their 5’ end at position -50 from the *atpA* start codon are highlighted with gray backgrounds.

**TABLE S2** Primers used in this work.

| Primer names | Sequences 5' to 3' |
| --- | --- |
| The following primers were used for detection of T-DNA insertion sites | |
| SAIL-LB2 | GCTTCCTATTATATCTTCCCAAATTACCAATACA |
| SALK-LBa1 | TGGTTCACGTAGTGGGCCATCG |
| BFA2-F | ATGGGTTCGTTAAGGTTTTCCA |
| BFA2-R | TATACCCATCTGTTTCATCCAC |
| BFA2-1030-F | GGTTTTCTCCGAATATCATTGC |
| BFA2-1588-F | TTTGCTCTTGCAAAGAATCTGG |
| BFA2-658-F | TCAACACGGTTATATATGCATG |
| BFA2-1258-R | ATGAATTGGGTTTATAACCACAC |
| CGL160-TDNA-F | CATCAGACATCAGGTGCGTCATAAG |
| CGL160-TDNA-R | CTTCCATTTAGGAGACACAATAGCC |
| SVR7-TDNA-F | CGGTAAACTCATTGGCTGTGTCGAGAA |
| SVR7-TDNA-R | CTACGCAGAAACTCCTCCAGCTGAG |
| The following primers were used for complementation vector construction | |
| BFA2-35S-F | GTATCTAGATTTGATGGGTTCGTTAAGG |
| BFA2-35S-R | AAGAGCTCCTATATACCCATCTGTTTCATCCAC |
| The following primers used for protein expression vectors construction | |
| BFA2-GFP-F | AATCTAGAATGGGTTCGTTAAGGTTTTCCA |
| BFA2-GFP-R | AACCGCGGCACAGCTCCTTGATCAGATCCT |
| BFA2-Ab-F | AAGGATCCGTCTCTAGTCTCGGTACTGAAGC |
| BFA2-Ab-R | GGCTCGAGTTCCTCTGCTTTATCATATAATCTC |
| BFA2-MBP-F | AACCATGGTCTCTAGTCTCGGTACTGAAGCA |
| BFA2- MBP-R | AAGAATTCTATACCCATCTGTTTCATCCAC |
| The following primers were used to prepare hybridization probes for Northern analysis | |
| ATPI-Northern-F | ATCATGTTCCATCAACACAC |
| ATPI-Northern-R | CCGCATAAAAATAGGCTAC |
| ATPH-Northern-F | ATGAATCCACTGGTTTCTG |
| ATPH-Northern-R | AAAGCGCTAATGCTACAAC |
| ATPF-Northern-F | TTAGATAACCGAAAGCAGAGG |
| ATPF-Northern-R | TTCATCGTACCAAACATCCC |
| ATPF-in-Northern-F | TCAAGAATAGGCTGGATTCACCCAG |
| ATPF-in-Northern-R | TTTGGCTCTCATGCTCACTTATTCC |
| ATPA-Northern-F | ATGGTAACCATTAGAGCCGAC |
| ATPA-Northern-R | TACTTTCTCCTGAAGTAGG |
| ATPB-Northern-F | AAATTGGACTATTCGGTGG |
| ATPB-Northern-R | CTTGAATAGAGGTTATGGACC |
| ATPE-Northern-F | AAATCTTTGTGTACTGACTCCG |
| ATPE-Northern-R | AAATCGTATTGAGAGCCTCG |
| ACT7-F | GGTGTCATGGTTGGTATGGGTC |
| ACT7-R | CCTCTGTGAGTAGAACTGGGTGC |
| The following primers were used for cRT-PCR | |
| atpA-F | CCGCTCAAATTAAAGCTATG |
| atpA-R | TCACCCATTAATACAACACC |
| atpA-RT | TGACTGAACTTCCTTCTTGG |
| atpF-RT | TCAATACACCGAAAACTACAC |
| atpF-F | ATCAAGTCCGCGAACGGG |
| atpF-R | GGTATTAAATCCGAAACTCCC |
| The following primers were used for EMSA analysis | |
| *atpF-atpA* | UAUAGGCAUUAUUUUUUUUUCU |
| *ndhA* | UUAUGACGAUACUCGGUAGCAUAGAUAUAA |
